# Platelets in Inflammation and Atherogenesis

**DOI:** 10.3389/fimmu.2015.00098

**Published:** 2015-03-06

**Authors:** Henry M. Nording, Peter Seizer, Harald F. Langer

**Affiliations:** ^1^University Clinic for Cardiology and Cardiovascular Medicine, Eberhard Karls-University Tübingen, Tübingen, Germany; ^2^Section for Cardioimmunology, Eberhard Karls-University Tübingen, Tübingen, Germany

**Keywords:** platelets, inflammation, atherosclerosis

## Abstract

Platelets contribute to processes beyond thrombus formation and may play a so far underestimated role as an immune cell in various circumstances. This review outlines immune functions of platelets in host defense, but also how they may contribute to mechanisms of infectious diseases. A particular emphasis is placed on the interaction of platelets with other immune cells. Furthermore, this article outlines the features of atherosclerosis as an inflammatory vascular disease highlighting the role of platelet crosstalk with cellular and soluble factors involved in atheroprogression. Understanding, how platelets influence these processes of vascular remodeling will shed light on their role for tissue homeostasis beyond intravascular thrombosis. Finally, translational implications of platelet-mediated inflammation in atherosclerosis are discussed.

## Introduction

The vasculature is one of the large networks of the human body and, thus, it needs to be well protected by immune mechanisms. When tissue is injured, the wound is paralleled by a severance of the vascular network, as well. Disruption of the endothelial monolayer lining all vessels from the inside triggers a process referred to as thrombus formation, a well regulated and complex cascade of events (1). During thrombus formation, other systems located within the vasculature can be activated, the most prominent of which is the immune system and inflammation being a part of its innate response. The inflammatory response to tissue injury triggers various events, which allow for defense against possible intruders and initiate the healing process (2). Simultaneously and in close proximity, platelets are recruited to the wound to restore endothelial integrity; they are activated and initiate thrombus formation. Given the close spatiotemporal relationship of these molecular processes, it is not surprising that growing evidence suggests that platelets are not only effectors of thrombus formation, but actively participate in inflammation and other processes related to tissue remodeling (3).

## Platelets Preserve Vascular Integrity

Repair of vascular damage while simultaneously preserving the patency of narrow capillaries is a complex task and requires a finely tuned machinery of pro- as well as anti-thrombotic mechanisms. Platelets are the key cells of primary hemostasis and thrombus formation. They mediate thrombus formation through cellular and soluble factors [recently also reviewed in Ref. (1, 4–7)]. GPIbα is a platelet transmembrane receptor associated with GPIX and GPV (8). GPIbα binding to von-Willebrand-factor (vWF) initiates primary hemostasis (8). In a shear-dependent fashion, GPIbα binding to vWF immobilized on collagen enables initial platelet rolling, which precedes all further steps of thrombus formation (9). Except at sites of high shear rates (10), stable platelet adhesion requires additional contribution of GPVI and integrins (11). GPVI is one of the platelet collagen receptors. It provides strong mechanic adhesion but also serves as the primary inducer of platelet activation mediated by its FcRγ-chain (12, 13). Amongst other signaling events, activation of GPVI causes an elevation of intracellular Ca^2+^ and subsequent platelet shape change (14). Platelet activation is paralleled by the secretion of soluble factors from platelet granules, the most important of which are ADP and TxA2 (15–17), as they activate platelets in an autocrine fashion (18). C-type lectin-like type II (CLEC-2) supports GPVI as it sustains a similar signaling pathway as the one induced by the GPVI/FcRγ complex (19, 20). In fact, CLEC-2 was found to be of particular importance for stable aggregate formation under flow conditions (21). The thrombotic activity of platelets is regulated by controlling the surface density of these major receptors by ectodomain shedding (22). A central process in platelet-mediated thrombus formation is integrin activation, as integrins connect the ECM to the platelets’ cytoskeleton and enable platelet aggregate formation (11). The integrin α_IIb_β_3_ has the ability to “crosslink” platelets via fibrinogen-bridging (23), thus stabilizing the forming thrombus. Due to its central importance, its inside-out activation is referred to as the “final common pathway of platelet activation” (24). The activation of α_IIb_β_3_ is mediated by the classical platelet agonists ADP or TxA2, and interfering with these pathways was successfully transferred into patient treatment (25). Similar to other integrins, α_IIb_β_3_ promotes “outside-in” signaling as well as platelet spreading and clot retraction (26). Finally, platelets also interact with the coagulation system in various ways stabilizing the developing thrombus by fibrin formation, which provides for provisional wound closure (27–29).

## “Non-Classical” Platelet Functions

Although traditionally not conceived as immune cells, platelets hold important functions in the immune response, particularly in innate immunity (30–32). In both host defense and preservation of vascular functions, platelets are helpful in some and harmful in other conditions (33). In the following, we will aim to exemplify how platelets mediate effects beyond thrombus formation.

### Platelets in host defense

Platelets contribute to pathogen recognition by interacting with immune cells, but also by interacting with the bacteria themselves (34–41). Recently, it was demonstrated that platelet-rich plasma (PRP) inhibited the growth of bacteria (42). The various receptor interactions involved in this platelet–bacteria crosstalk were already reviewed elsewhere (43, 44). For instance, platelets are able to recognize CpG islands upon thrombin activation and subsequent TLR9 expression (40). Furthermore, platelets react to fungal infections *in vitro* and *in vivo* (45). Finally, platelet “nuclear functions” are increasingly uncovered and recognized (2). Via transcription of mRNA and post-transcriptional modification (46–48), platelets seem to contribute to the inflammasome by producing IL-1β (49), they are involved in modulating NFκB (50) and may influence endothelial polarization by miRNA secretion (51).

### Platelets contributing to mechanisms of infections

On the other hand, there are a number of reports describing platelets as an important element in the progression of infections (52). The platelet receptor CLEC-2 has been shown to facilitate the entry of HI-viruses (53), and platelets contribute to disease progression via CD40L (54, 55). Furthermore, platelets are involved in the progression of HBV-infection and other viral diseases (56, 57) by the recruitment of cytotoxic T-cells (CTL) to the liver (58) or other organs in a serotonin-dependent manner (59). Verschoor et al. could recently show that platelets are a relevant factor in the process of immune evasion by intracellular bacteria such as *Listeria* (38). Furthermore, platelets play an important role in infections by *Leishmania* (60) and in the pathogenesis of Hantavirus infection (61). A fact, which complicates the picture even more, is that platelets can also modulate the function of further cells involved in the response to infections – the leukocyte.

### Platelet crosstalk with immune cells

One of the main immune mechanisms of platelets is their capability to recruit leukocytes to sites of infection and inflammation (32, 62). P-selectin–PSGL-1 binding (63, 64), ICAM1 (51), and GPIbα (65) play an important role in how platelets bring other immune cells to the scene (66), particularly under high shear conditions (67). Platelets have the ability to form aggregates with neutrophils (68). In periodontitis, aggregate formation of platelets and neutrophils (NPA) enhances neutrophil phagocytosis in a TLR-2-dependent manner (69). In acute lung injury, NPA formation mediates neutrophil extravasation (70) and platelet-derived platelet factor 4 (PF4) fostered neutrophil survival in a model of arterial occlusion (71). Furthermore, platelet–leukocyte aggregates can be used as a diagnostic tool, for example as a parameter to assess sepsis severity (72). Another recently discovered way, how platelets modulate neutrophil function is their involvement in neutrophil extracellular trap (NET) formation to ensnare intruders (73). Platelet TLR-4 (74) as well as platelet β-defensins have been implicated in NET formation (75, 76). Specifically, platelet-induced NET formation may play a role in viral infections (77) or transfusion-related lung injury (78). Rossaint et al. have recently proposed that simultaneous activation of neutrophils via Mac-1 outside-in signaling and Gαi engagement by platelet-derived RANTES–PF4 heterodimers is required for NET formation (79). Interestingly, platelets seem to form especially stable aggregates with monocytes (80), and activated platelets induce an inflammatory monocyte phenotype (81). As this process seems to be partially independent of P-selectin interaction with PSGL-1, paracrine mechanisms to strengthen platelet–monocyte aggregate formation have been proposed as an alternative mechanism (81). Platelet–monocyte interaction seems to be of functional relevance, as their formation increases the number of circulating monocytes with a higher affinity for adhesion to the endothelium (82). Furthermore, activated platelets are taken up by monocytes which induces enhancement of cytokine release from macrophages (83). Other authors, however, report on anti-inflammatory effects of platelet–monocyte interaction (84–86) via CXCR5 engagement of CXCL13 on monocytes (84) or, following experimental sepsis, by inhibition of macrophage tumor necrosis factor α (TNF-α) and IL-6 secretion (85, 86). Thus, the effect of platelets on monocytes appears to be context-dependent.

Via P-selectin PSGL-1 interaction, platelets can form aggregates with lymphocytes (PLA), as well. Platelet interaction with T-cells, B-cells, and NK-cells induces their homing, activation, and recruitment as recently reviewed (87). Platelets may even serve as a bridge directing T-cells to the endothelium (88). Furthermore, platelets modulate lymphocyte function via direct cell–cell interaction as well as soluble mediators (87). In rheumatoid arthritis patients, platelet binding to lymphocytes promoted activation-induced proliferation as well as IL-17 and interferon-γ production by GPVI positive CD4+ T-cells (89). Serotonin from platelet vesicles may also stimulate T-lymphocytes (90). Through release of PF4 and CCL5, platelets can enhance cytokine production in CD4+ T-cells (34). Furthermore, in HCV infection, platelet CCL5 causes upregulation of T-lymphocyte helper cells type 1 (Th1) (91). Finally, platelets may also enhance T-cell-mediated germinal center formation and release of specific IgGs from B-cells via CD40L signaling (35, 41). In fact, platelets may substitute CD40L when few CD40L-positive T-cells are present to stimulate B-cell maturation (92). However, platelets may also induce anti-inflammatory effects. PF4 released from platelets leads to an increase in regulatory T-lymphocytes (Tregs) (93) and limits Th17 differentiation (94). Interestingly, T-lymphocytes can activate platelets, which amplify the release of CCL5 (95). Addressing antigen-presenting cells, there are a number of ways in which platelets interact with dendritic cells (DCs). For instance, platelets can recruit DC via Mac-1 interaction with JAM-C and can activate them inducing platelet phagocytosis and subsequently apoptosis of DCs (96). High shear rates may be a trigger for platelets to recruit DCs and promote their maturation (97). In fact, direct contact of platelets and DCs seems to induce other effects than crosstalk via soluble factors suggesting that platelets have the ability to differentially regulate a DC response (98). This conclusion is supported by recent findings demonstrating that platelets can enhance DC-mediated Th-2 cell response in allergy by secreted RANKL (99). Platelets can, however, also impair DC differentiation or reduce DC production of proinflammatory cytokines IL-12p70 and TNF-α (100).

## Platelets in Atherosclerosis

**Figure 1:**
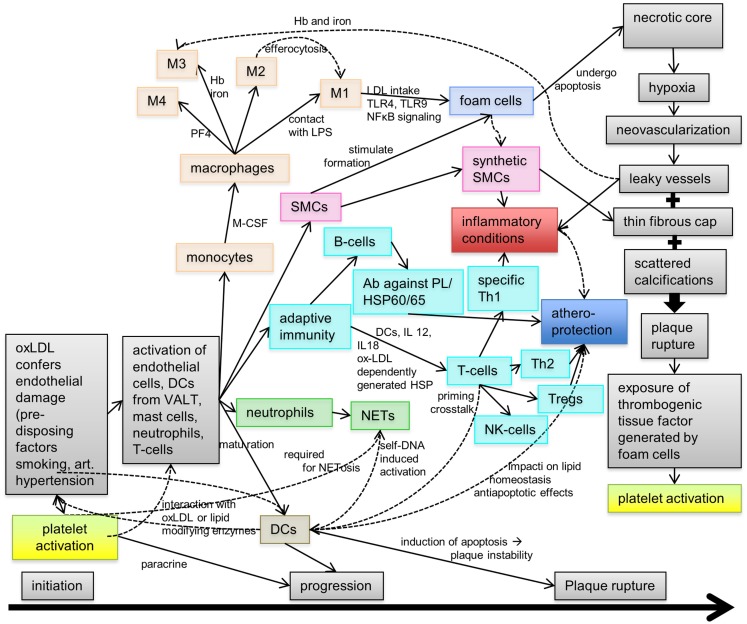
**Pathophysiology of atherosclerosis**. During atherogenesis, a plaque forms on the luminal side of the arterial wall through a complex process involving lipid accumulation, cellular activation inducing the transformation and differentiation of monocytes into foam cells, and various immune reactions mediated by T- and B-cells, neutrophils, granulocytes (neutrophils), and dendritic cells (DCs). Hence, the progression of atherosclerosis is driven by inflammation, although some of these inflammatory cells/factors may also mediate atheroprotection under certain conditions. At later stages, the atherosclerotic plaque develops a necrotic core with areas of neovascularization, a thin fibrous cap, and scattered calcifications. Finally, plaque rupture exposes the thrombogenic atherosclerotic core inducing platelet activation and, subsequently, initiation of the coagulation cascade. Ab, antibody; Hb, hemoglobin; HSP, heat shock protein; NET, neutrophil extracellular trap; PF4, platelet factor 4; SMC, smooth muscle cell; Treg, regulatory T-cell; VALT, vascular-associated lymphatic tissue.

Atherosclerosis is a chronic inflammatory disease featuring various complex processes contributing to its pathophysiology and the development of the atherosclerotic plaque over decades. Figure 1 summarizes the steps contributing to fatty streak formation, inflammation, progression of the plaque, and finally plaque rupture.

### Platelet contribution to atherosclerosis

**Figure 2:**
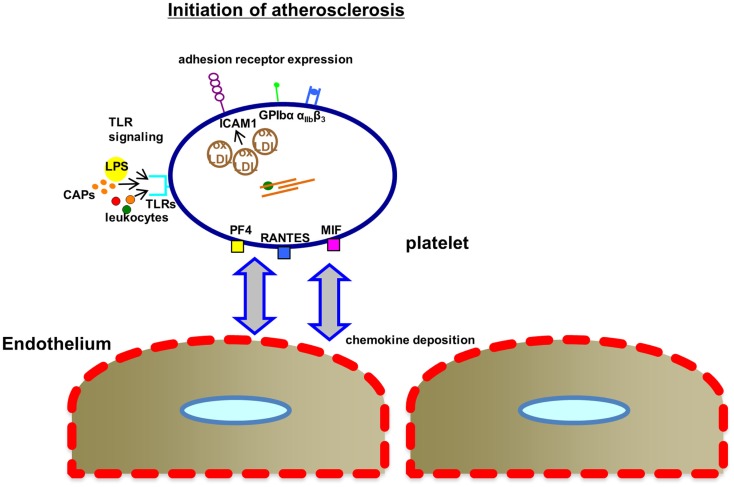
**Mechanisms, how platelets affect the initiation of atherosclerosis**. Upon activation within the microenvironment of the atherosclerotic plaque or even at very early stages of atherogenesis in areas of high shear stress, platelets express increased levels of inflammatory adhesion receptors (ICAM1, intracellular adhesion molecule 1; GPIbα, glycoprotein Ibα; α_IIb_β_3_, glycoprotein α_IIb_β_3_) associated with oxLDL binding to platelets. TLR-mediated signaling may contribute via binding of lipopolysaccharides (LPS), leukocyte interaction, or binding of carboxyalkylpyrroles (CAPs). The complement system also contributes to atherogenesis. MIF, macrophage migratory inhibitory factor; PF4, platelet factor 4; RANTES, chemokine ligand 5 (CCL5); TLRs, Toll-like receptors; oxLDL, oxidized low-density lipoprotein.

**Figure 3:**
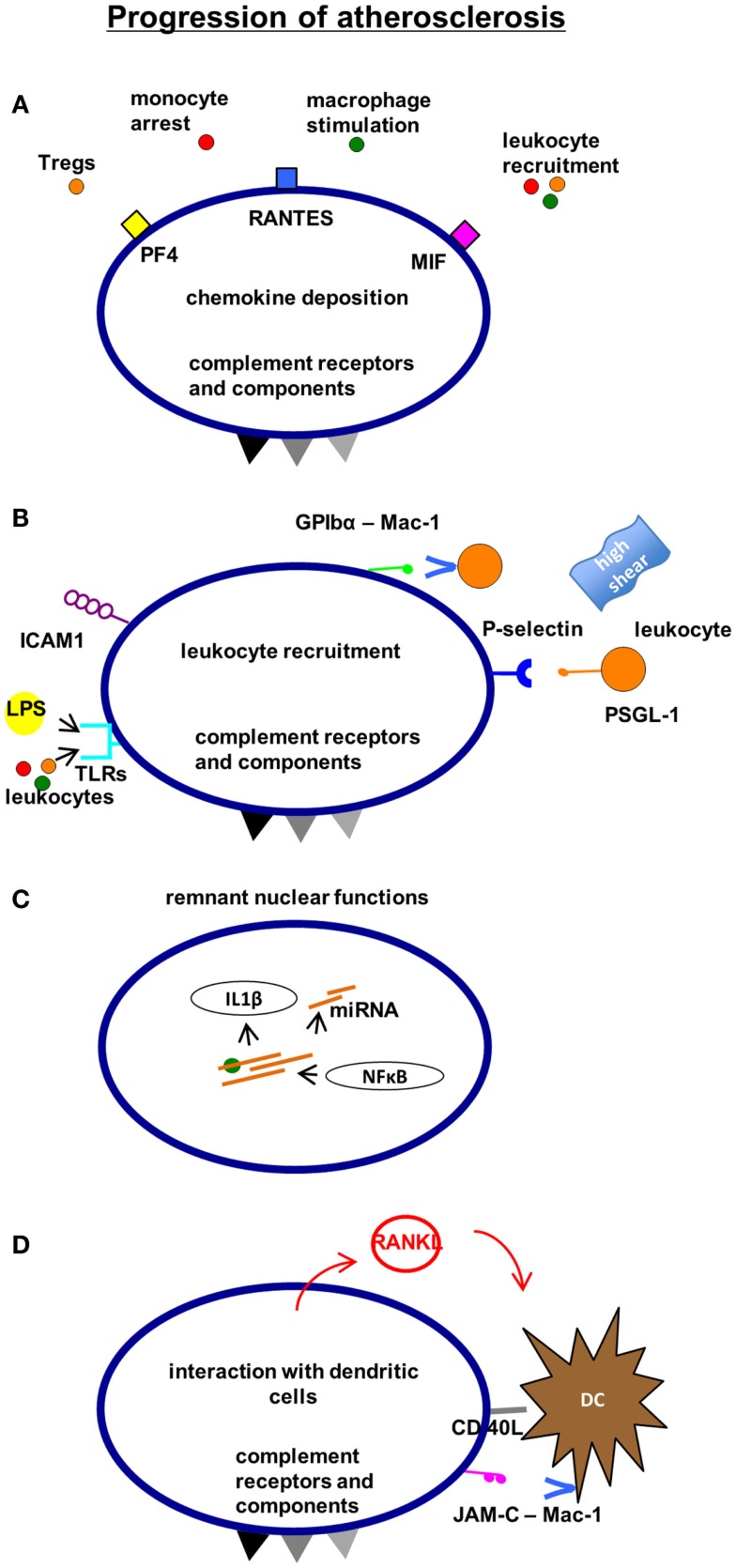
**Mechanisms, how platelets affect the progression of atherosclerosis**. Platelets contribute to the progression of atherosclerosis by chemokine deposition **(A)** and leukocyte recruitment **(B)**. Remnant “nuclear” functions of platelets such as the ability to translate and modify mRNA promote inflammation and endothelial polarization **(C)**. Furthermore, the interaction with antigen-presenting dendritic cells can contribute to atheroprogression involving adhesion receptors and soluble mediators **(D)**. CXCR4, C-X-C chemokine receptor type 4 (CD184); SDF-1α, stromal cell-derived factor 1α.

**Figure 4:**
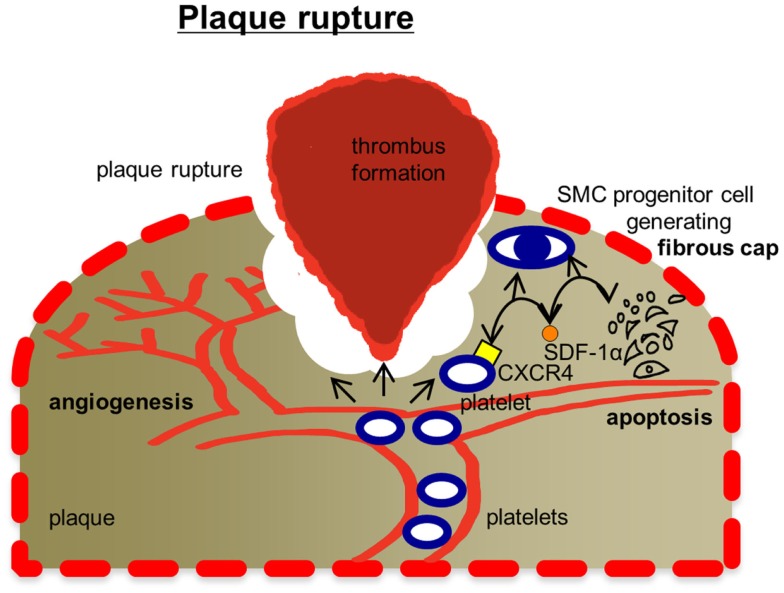
**Mechanisms, how platelets affect plaque rupture**. Plaque rupture is tailored by platelets in several ways: platelets are known to modulate angiogenesis, which is a decisive factor for plaque stability. Platelets have also been found to promote smooth muscle cell (SMC) progenitor recruitment via CXCR4-SDF-1α signaling. SMCs are responsible for generating extracellular matrix stabilizing the fibrous cap of the plug. Thrombus formation in areas of the ruptured fibrous cap exposing components of the extracellular matrix is initiated by platelets. Future investigations will also have to test, whether platelets enter the plaque tissue and affect tissue remodeling. Ab, antibody; CD40L, CD40 ligand (CD154); DC, dendritic cell; IL-1α, interleukin 1α; JAM-C, junctional adhesion molecule type C; Mac-1, macrophage-1 antigen; MIF, macrophage migratory inhibitory factor; miRNA, microRNA; NFκB, nuclear factor kappa-light-chain-enhancer of activated B-cells; PF4, platelet factor 4; PSGL-1, P-selectin glycoprotein ligand-1; RANKL, receptor activator of nuclear factor kappa-B ligand; RANTES, chemokine ligand 5 (CCL5); TLRs, Toll-like receptors; Treg, regulatory T-cell.

In atherosclerosis, platelets are known to contribute to early steps of this chronic vascular pathology (Figure 2) such as endothelial dysfunction (101, 102), but also to final events such as rupture of the vulnerable plaque [(103), see also Figure 4]. For instance, platelets participate in atherogenesis by chemokine release (79, 104, 105), surface association of oxLDL (106), direct cell–cell interaction (107, 108), release of microparticles (109), and provision of inflammatory mediators [(110), see also Figure 3]. Platelets within the atherosclerotic plaque may remain activated for a long time providing for proinflammatory IL-1β production [(111), see also Figure 3]. One of the most considered functions of platelets in atherosclerosis is the recruitment of leukocytes via direct receptor–ligand interactions or augmented by released factors such as chemokines [(112, 113), see also previous sections and Figure 3]. The role of a particular leukocyte subtype – DCs, the classical antigen presenting cells of our body – has been emphasized recently in the context of atherosclerosis and, interestingly, platelets interact with DCs (Figure 3). In fact, GPIb–Mac-1 interaction may be an interesting signaling mechanism in the context of platelet–DC crosstalk modulating atheroprogression (114, 115). This is of particular importance, as DCs have been proposed to play a significant role in the different steps of atherosclerosis (116).

### Platelets in the complement system and atherosclerosis

A further part of our innate immune response, the complement system receives increasing attention in the context of atherosclerosis. This cascade of soluble plasma proteins constitutes a phylogenetically very old part of the inherited immune system (117). Complement activation is important for inflammatory conditions associated with vascular injury (118, 119). Most interestingly, platelets were reported to express a number of complement receptors relevant for platelet function and their crosstalk with the local microenvironment (120–122). Several complement components can be bound to the platelet surface (123, 124). We found that expression of complement anaphylatoxin receptors on platelets showed a strong and positive correlation with platelet activation markers such as P-selectin in patients with atherosclerosis (125). Further mechanistic studies will have to address the relevance of this association. In a recent review, the literature on platelets and potential intersection points with the complement system in diverse settings was summarized (126). Additional profound studies are needed to differentiate our understanding of the intersection points of platelet activation with the immunological elements of atherogenesis such as endothelial inflammation, leukocyte recruitment, antigen presentation, chemokine and cytokine production, or complement activation.

### Plaque rupture

At later stages, the atherosclerotic core becomes hypoxic inducing the outgrowth of vasa vasorum from the adventitia toward the intima [(127, 128), see also Figure 4]. As a consequence, fragile and leaky vessels form, which facilitate further invasion of immune cells and release of soluble factors into the surrounding atherosclerotic tissue (129, 130). Moreover, red blood cells get stuck in the plaque liberating hemoglobin and iron (131). These mechanisms ultimately result in plaque destabilization (132). Foam cells produce tissue factor (133), and as soon as the thrombogenic lipid core is exposed to the lumen, fibrin generation is initiated (134–136). In parallel, platelets as well as the coagulation cascade become activated (137, 138). Plaque rupture in the region of a thin fibrous cap is the final event, how atherosclerosis causes acute vascular complications such as myocardial infarction or stroke (139–143).

### Translational relevance

The fact that inflammation plays a key role in central steps of atherosclerosis (31, 144), is increasingly integrated into clinical considerations. Accordingly, this hypothesis is addressed by two current trials, the CANTOS trial launched in 2011 (145) and the CIRT trial (146) using immunosuppressants to treat atherosclerosis. Earlier in this article, we have aimed at depicting the importance of platelets for inflammation in atherosclerosis. Inhibition of platelet-mediated inflammation may already be everyday clinical practice considering the use of aspirin for the treatment of cardiovascular disease (147). As an irreversible inhibitor of the enzyme cyclooxygenase, aspirin is a mild inhibitor of platelet function (148). Despite its widespread use, the definite role of aspirin in the prevention of atherosclerosis and atherosclerosis-related diseases is still under discussion (147). There is evidence from preclinical studies that aspirin is able to inhibit the initiation (149) and even the progression of experimental atherosclerosis (150) via its effect on prostaglandin synthesis but also by other mechanisms such as the modulation of endothelial NO synthesis (151), NFκB signaling (152), CRP, or soluble CD40 ligand (sCD40L) (153). Clinical studies on the use of aspirin for primary prevention of atherosclerosis, however, have also yielded negative results [recently reviewed by Gaziano and Greenland (147)]. A large study in a Japanese population over 60 years of age could demonstrate no benefit of low-dose aspirin therapy (154, 155). In patients at low risk for cardiovascular events, the use of aspirin needs to be weighed very carefully against an elevated risk of bleeding events or even hemorrhagic stroke (147).

For the ADP-receptor antagonist clopidogrel, reports on an effect in the context of atherosclerosis exist, too. In animal models, clopidogrel has the ability to slow down the inflammatory progression of atherosclerosis (156, 157). Clopidogrel reduces platelet activation as measured by P-selectin expression and other inflammatory markers (158), while others stress that important inflammatory markers such as hsCRP are not affected (159, 160). On the other hand, platelet–leukocyte aggregate formation is inhibited more effectively by clopidogrel compared to aspirin (161, 162). In contrast, another group reported that under therapy with clopidogrel, the expression of some inflammatory chemokines may even be increased in peripheral blood mononuclear cells in patients with coronary artery disease (163). On the platelet surface, a number of inflammatory receptors may represent potential targets for new therapeutic approaches such as CXCL4, CCL5, CD40 ligand, PSGL-1 (164). Further targets may be platelet-activating factor (PAF) (165) or Annexin A5 (166). Finally, we have already described the evidence on complement receptor involvement in atherosclerosis (126, 167). This class of receptors are involved in a large number of inflammatory processes (117) and are also expressed on platelets (120–122). There are a number of substances targeting different parts of the complement system which are evaluated in different stages of clinical trials in conditions such as age-related macular degeneration or hereditary angioedema (168, 169). Some have even established themselves as first-line treatment such as eculizumab for paroxysmal nocturnal hemoglobinuria (170, 171). It is tempting to speculate, that these substances might be worth an evaluation in the context of atherosclerosis, as well.

Moreover, biomarkers of atherosclerosis are of great importance from a clinical point of view. Due to the high prevalence of cardiovascular disease, it is vital to identify which patient is at particular risk for adverse cardiovascular events and would benefit from preventive diagnostic or therapeutic interventions. A number of platelet surface receptors may be promising candidates to consider in this context. A prominent example is soluble CD40 ligand (sCD40L) released from platelets (172). In a number of settings such as on hospital admission of patients with acute coronary syndrome or in patients undergoing primary angioplasty, sCD40L levels appear to have predictive capacity (173–175). Some authors even discuss sCD40L as a therapeutic target (176). Apart from sCD40L, soluble P-selectin released from platelets is referred to as a further potential platelet-derived biomarker (177, 178).

Considering the achievements of platelet research over the last two decades with a bounty of platelet-targeted drugs, which found their way into everyday clinical practice, platelets and platelet-associated molecular mechanisms offer potential translational applications.

## Conclusion

In conclusion, platelets – conceived as immune cells and mediators of vascular/tissue remodeling – have a strong impact on atherosclerosis through inflammatory mechanisms discussed here on the basis of selected cellular or soluble platelet-derived mediators. The net effect of platelet-mediated inflammation may be an atheropromoting one, although these anucleate cells may mediate distinct atheroprotective mechanisms, as well. Future investigations will have to identify these specific platelet aspects to enable us to develop better diagnostic markers and therapeutic approaches with fewer undesired side effects.

## Conflict of Interest Statement

The authors declare that the research was conducted in the absence of any commercial or financial relationships that could be construed as a potential conflict of interest.

## References

[1] Jackson SP. Arterial thrombosis – insidious, unpredictable and deadly. Nat Med (2011) 17(11):1423–36. 10.1038/nm.2515 22064432

[2] Rondina MT Weyrich AS Zimmerman GA. Platelets as cellular effectors of inflammation in vascular diseases. Circ Res (2013) 112(11):1506–19. 10.1161/CIRCRESAHA.113.300512 23704217 PMC3738064

[3] Langer HF Weber C Gawaz M The platelet – thrombosis and beyond. Thromb Haemost (2013) 110(5):857–8 10.1160/TH13-09-0805 24108387

[4] Hagedorn I Vögtle T Nieswandt B. Arterial thrombus formation. Novel mechanisms and targets. Hämostaseologie (2010) 30(3):127–35. 20680230

[5] Nieswandt B Pleines I Bender M. Platelet adhesion and activation mechanisms in arterial thrombosis and ischaemic stroke. J Thromb Haemost (2011) 9(s1):92–104. 10.1111/j.1538-7836.2011.04361.x 21781245

[6] Berndt M Metharom P Andrews R. Primary haemostasis: newer insights. Haemophilia (2014) 20(s4):15–22. 10.1111/hae.12427 24762270

[7] Etulain J Schattner M. Glycobiology of platelet-endothelial cell interactions. Glycobiology (2014) 24(12):1252–9. 10.1093/glycob/cwu056 24928621

[8] Andrews RK Berndt MC The glycoprotein Ib-IX-V complex. In: Michelson A, editor. Platelets. 3rd ed San Diego: Academic Press (2012). p. 195–212.

[9] Bergmeier W Piffath CL Goerge T Cifuni SM Ruggeri ZM Ware J The role of platelet adhesion receptor GPIbα far exceeds that of its main ligand, von Willebrand factor, in arterial thrombosis. Proc Natl Acad Sci U S A (2006) 103(45):16900–5. 10.1073/pnas.0608207103 17075060 PMC1636551

[10] Nesbitt WS Westein E Tovar-Lopez FJ Tolouei E Mitchell A Fu J A shear gradient-dependent platelet aggregation mechanism drives thrombus formation. Nat Med (2009) 15(6):665–73. 10.1038/nm.1955 19465929

[11] Nieswandt B Varga-Szabo D Elvers M. Integrins in platelet activation. J Thromb Haemost (2009) 7(s1):206–9. 10.1111/j.1538-7836.2009.03370.x 19630801

[12] Moroi M Jung SM. Platelet glycoprotein VI: its structure and function. Thromb Res (2004) 114(4):221–33. 10.1016/j.thromres.2004.06.046 15381385

[13] Bergmeier W Stefanini L. Platelet ITAM signaling. Curr Opin Hematol (2013) 20(5):445–50. 10.1097/MOH.0b013e3283642267 23921514

[14] Stefanini L Boulaftali Y Ouellette TD Holinstat M Désiré L Leblond B Rap1-Rac1 circuits potentiate platelet activation. Arterioscler Thromb Vasc Biol (2012) 32(2):434–41. 10.1161/ATVBAHA.111.239194 22075250 PMC3262085

[15] Moers A Nieswandt B Massberg S Wettschureck N Grüner S Konrad I G13 is an essential mediator of platelet activation in hemostasis and thrombosis. Nat Med (2003) 9(11):1418–22. 10.1038/nm943 14528298

[16] Huang J-S Ramamurthy SK Lin X Le Breton GC. Cell signalling through thromboxane A2 receptors. Cell Signal (2004) 16(5):521–33. 10.1016/j.cellsig.2003.10.008 14751539

[17] Jin J Daniel JL Kunapuli SP. Molecular basis for ADP-induced platelet activation II. The P2Y1 receptor mediates ADP-induced intracellular calcium mobilization and shape change in platelets. J Biol Chem (1998) 273(4):2030–4. 10.1074/jbc.273.4.2030 9442040

[18] Offermanns S. Activation of platelet function through G protein-coupled receptors. Circ Res (2006) 99(12):1293–304. 10.1161/01.RES.0000251742.71301.16 17158345

[19] Fuller GL Williams JA Tomlinson MG Eble JA Hanna SL Pöhlmann S The C-type lectin receptors CLEC-2 and Dectin-1, but not DC-SIGN, signal via a novel YXXL-dependent signaling cascade. J Biol Chem (2007) 282(17):12397–409. 10.1074/jbc.M609558200 17339324 PMC1997429

[20] Suzuki-Inoue K Fuller GL García Á Eble JA Pöhlmann S Inoue O A novel Syk-dependent mechanism of platelet activation by the C-type lectin receptor CLEC-2. Blood (2006) 107(2):542–9. 10.1182/blood-2005-05-1994 16174766

[21] May F Hagedorn I Pleines I Bender M Vögtle T Eble J CLEC-2 is an essential platelet-activating receptor in hemostasis and thrombosis. Blood (2009) 114(16):3464–72. 10.1182/blood-2009-05-222273 19641185

[22] Gardiner E Karunakaran D Shen Y Arthur J Andrews R Berndt M. Controlled shedding of platelet glycoprotein (GP) VI and GPIb-IX-V by ADAM family metalloproteinases. J Thromb Haemost (2007) 5(7):1530–7. 10.1111/j.1538-7836.2007.02590.x 17445093

[23] Gawaz MP Loftus J Bajt M Frojmovic M Plow E Ginsberg M. Ligand bridging mediates integrin alpha IIb beta 3 (platelet GPIIB-IIIA) dependent homotypic and heterotypic cell-cell interactions. J Clin Invest (1991) 88(4):1128. 10.1172/JCI115412 1918367 PMC295567

[24] Varga-Szabo D Pleines I Nieswandt B. Cell adhesion mechanisms in platelets. Arterioscler Thromb Vasc Biol (2008) 28(3):403–12. 10.1161/ATVBAHA.107.150474 18174460

[25] Ozaki Y Asazuma N Suzuki-Inoue K Berndt MC. Platelet GPIb-IX-V-dependent signaling. J Thromb Haemost (2005) 3(8):1745–51. 10.1111/j.1538-7836.2005.01379.x 16102041

[26] Naik UP Naik MU. Association of CIB with GPIIb/IIIa during outside-in signaling is required for platelet spreading on fibrinogen. Blood (2003) 102(4):1355–62. 10.1182/blood-2003-02-0591 12714504

[27] Munnix IC Kuijpers MJ Auger J Thomassen CM Panizzi P van Zandvoort MA Segregation of platelet aggregatory and procoagulant microdomains in thrombus formation regulation by transient integrin activation. Arterioscler Thromb Vasc Biol (2007) 27(11):2484–90. 10.1161/ATVBAHA.107.151100 17761939 PMC2376762

[28] Müller F Mutch NJ Schenk WA Smith SA Esterl L Spronk HM Platelet polyphosphates are proinflammatory and procoagulant mediators in vivo. Cell (2009) 139(6):1143–56. 10.1016/j.cell.2009.11.001 20005807 PMC2796262

[29] Kleinschnitz C Stoll G Bendszus M Schuh K Pauer H-U Burfeind P Targeting coagulation factor XII provides protection from pathological thrombosis in cerebral ischemia without interfering with hemostasis. J Exp Med (2006) 203(3):513–8. 10.1084/jem.20052458 16533887 PMC2118228

[30] Semple JW Italiano JE Freedman J. Platelets and the immune continuum. Nat Rev Immunol (2011) 11(4):264–74. 10.1038/nri2956 21436837

[31] Libby P Ridker PM Hansson GK. Inflammation in atherosclerosis: from pathophysiology to practice. J Am Coll Cardiol (2009) 54(23):2129–38. 10.1016/j.jacc.2009.09.009 19942084 PMC2834169

[32] Herter JM Rossaint J Zarbock A. Platelets in inflammation and immunity. J Thromb Haemost (2014) 12(11):1764–75. 10.1111/jth.12730 25224706

[33] von Hundelshausen P Weber C. Platelets as immune cells bridging inflammation and cardiovascular disease. Circ Res (2007) 100(1):27–40. 10.1161/01.RES.0000252802.25497.b7 17204662

[34] Gerdes N Zhu L Ersoy M Hermansson A Hjemdahl P Hu H Platelets regulate CD4(+) T-cell differentiation via multiple chemokines in humans. Thromb Haemost (2011) 106(2):353–62. 10.1160/TH11-01-0020 21655676

[35] Elzey BD Grant JF Sinn HW Nieswandt B Waldschmidt TJ Ratliff TL. Cooperation between platelet-derived CD154 and CD4+ T cells for enhanced germinal center formation. J Leukoc Biol (2005) 78(1):80–4. 10.1189/jlb.1104669 15899982

[36] Sprague DL Elzey BD Crist SA Waldschmidt TJ Jensen RJ Ratliff TL. Platelet-mediated modulation of adaptive immunity: unique delivery of CD154 signal by platelet-derived membrane vesicles. Blood (2008) 111(10):5028–36. 10.1182/blood-2007-06-097410 18198347 PMC2384131

[37] Zhang X Liu Y Gao Y Dong J Mu C Lu Q Inhibiting platelets aggregation could aggravate the acute infection caused by *Staphylococcus aureus*. Platelets (2011) 22(3):228–36. 10.3109/09537104.2010.543962 21265599

[38] Verschoor A Neuenhahn M Navarini AA Graef P Plaumann A Seidlmeier A A platelet-mediated system for shuttling blood-borne bacteria to CD8 [alpha]+ dendritic cells depends on glycoprotein GPIb and complement C3. Nat Immunol (2011) 12(12):1194–201. 10.1038/ni.2140 22037602

[39] Cognasse F Hamzeh-Cognasse H Lafarge S Delezay O Pozzetto B McNicol A Toll-like receptor 4 ligand can differentially modulate the release of cytokines by human platelets. Br J Haematol (2008) 141(1):84–91. 10.1111/j.1365-2141.2008.06999.x 18279456

[40] Thon JN Peters CG Machlus KR Aslam R Rowley J Macleod H T granules in human platelets function in TLR9 organization and signaling. J Cell Biol (2012) 198(4):561–74. 10.1083/jcb.201111136 22908309 PMC3514030

[41] Elzey BD Tian J Jensen RJ Swanson AK Lees JR Lentz SR Platelet-mediated modulation of adaptive immunity. A communication link between innate and adaptive immune compartments. Immunity (2003) 19(1):9–19. 10.1016/S1074-7613(03)00177-8 12871635

[42] Mariani E Filardo G Canella V Berlingeri A Bielli A Cattini L Platelet-rich plasma affects bacterial growth in vitro. Cytotherapy (2014) 16(9):1294–304. 10.1016/j.jcyt.2014.06.003 25108654

[43] Fitzgerald JR Foster TJ Cox D. The interaction of bacterial pathogens with platelets. Nat Rev Microbiol (2006) 4(6):445–57. 10.1038/nrmicro1425 16710325

[44] Yeaman MR. Platelets in defense against bacterial pathogens. Cell Mol Life Sci (2010) 67(4):525–44. 10.1007/s00018-009-0210-4 20013024 PMC2809947

[45] Speth C Rambach G Lass-Flörl C. Platelet immunology in fungal infections. Thromb Haemost (2014) 112(4):632–9. 10.1160/TH14-01-0074 24990293

[46] Weyrich AS Denis MM Schwertz H Tolley ND Foulks J Spencer E mTOR-dependent synthesis of Bcl-3 controls the retraction of fibrin clots by activated human platelets. Blood (2007) 109(5):1975–83. 10.1182/blood-2006-08-042192 17110454 PMC1801071

[47] Rondina M Schwertz H Harris E Kraemer B Campbell R Mackman N The septic milieu triggers expression of spliced tissue factor mRNA in human platelets. J Thromb Haemost (2011) 9(4):748–58. 10.1111/j.1538-7836.2011.04208.x 21255247 PMC3071458

[48] Schwertz H Tolley ND Foulks JM Denis MM Risenmay BW Buerke M Signal-dependent splicing of tissue factor pre-mRNA modulates the thrombogenicity of human platelets. J Exp Med (2006) 203(11):2433–40. 10.1084/jem.20061302 17060476 PMC2118136

[49] Brown GT McIntyre TM. Lipopolysaccharide signaling without a nucleus: kinase cascades stimulate platelet shedding of proinflammatory IL-1β-rich microparticles. J Immunol (2011) 186(9):5489–96. 10.4049/jimmunol.1001623 21430222 PMC3100655

[50] Gambaryan S Kobsar A Rukoyatkina N Herterich S Geiger J Smolenski A Thrombin and collagen induce a feedback inhibitory signaling pathway in platelets involving dissociation of the catalytic subunit of protein kinase A from an NFκB-IκB complex. J Biol Chem (2010) 285(24):18352–63. 10.1074/jbc.M109.077602 20356841 PMC2881761

[51] Gidlöf O van der Brug M Öhman J Gilje P Olde B Wahlestedt C Platelets activated during myocardial infarction release functional miRNA, which can be taken up by endothelial cells and regulate ICAM1 expression. Blood (2013) 121(19):3908–17. 10.1182/blood-2012-10-461798 23493781

[52] Assinger A. Platelets and infection – an emerging role of platelets in viral infection. Front Immunol (2014) 5:649. 10.3389/fimmu.2014.00649 25566260 PMC4270245

[53] Chaipan C Soilleux EJ Simpson P Hofmann H Gramberg T Marzi A DC-SIGN and CLEC-2 mediate human immunodeficiency virus type 1 capture by platelets. J Virol (2006) 80(18):8951–60. 10.1128/JVI.00136-06 16940507 PMC1563896

[54] Pate KAM Lyons CE Dorsey JL Shirk EN Queen SE Adams RJ Platelet activation and platelet-monocyte aggregate formation contribute to decreased platelet count during acute simian immunodeficiency virus infection in pig-tailed macaques. J Infect Dis (2013) 208(6):874–83. 10.1093/infdis/jit278 23852120 PMC3749014

[55] Singh MV Davidson DC Jackson JW Singh VB Silva J Ramirez SH Characterization of platelet-monocyte complexes in hiv-1-infected individuals: possible role in HIV-associated neuroinflammation. J Immunol (2014) 192(10):4674–84. 10.4049/jimmunol.1302318 24729609 PMC4011982

[56] Iannacone M Sitia G Narvaiza I Ruggeri ZM Guidotti LG. Antiplatelet drug therapy moderates immune-mediated liver disease and inhibits viral clearance in mice infected with a replication-deficient adenovirus. Clin Vaccine Immunol (2007) 14(11):1532–5. 10.1128/CVI.00298-07 17881509 PMC2168169

[57] Iannacone M Sitia G Isogawa M Whitmire JK Marchese P Chisari FV Platelets prevent IFN-α/β-induced lethal hemorrhage promoting CTL-dependent clearance of lymphocytic choriomeningitis virus. Proc Natl Acad Sci U S A (2008) 105(2):629–34. 10.1073/pnas.0711200105 18184798 PMC2206587

[58] Iannacone M Sitia G Isogawa M Marchese P Castro MG Lowenstein PR Platelets mediate cytotoxic T lymphocyte-induced liver damage. Nat Med (2005) 11(11):1167–9. 10.1038/nm1317 16258538 PMC2908083

[59] Lang PA Contaldo C Georgiev P El-Badry AM Recher M Kurrer M Aggravation of viral hepatitis by platelet-derived serotonin. Nat Med (2008) 14(7):756–61. 10.1038/nm1780 18516052

[60] Goncalves R Zhang X Cohen H Debrabant A Mosser DM. Platelet activation attracts a subpopulation of effector monocytes to sites of *Leishmania* major infection. J Exp Med (2011) 208(6):1253–65. 10.1084/jem.20101751 21606505 PMC3173254

[61] Vaheri A Strandin T Hepojoki J Sironen T Henttonen H Makela S Uncovering the mysteries of hantavirus infections. Nat Rev Microbiol (2013) 11(8):539–50. 10.1038/nrmicro3066 24020072

[62] Zarbock A Polanowska-Grabowska RK Ley K. Platelet-neutrophil-interactions: linking hemostasis and inflammation. Blood Rev (2007) 21(2):99–111. 10.1016/j.blre.2006.06.001 16987572

[63] Frenette PS Denis CV Weiss L Jurk K Subbarao S Kehrel B P-selectin glycoprotein ligand 1 (PSGL-1) is expressed on platelets and can mediate platelet-endothelial interactions in vivo. J Exp Med (2000) 191(8):1413–22. 10.1084/jem.191.8.1413 10770806 PMC2193129

[64] Lam FW Burns AR Smith CW Rumbaut RE. Platelets enhance neutrophil transendothelial migration via P-selectin glycoprotein ligand-1. Am J Physiol Heart Circ Physiol (2011) 300(2):H468–75. 10.1152/ajpheart.00491.2010 21169400 PMC3044064

[65] Wong CH Jenne CN Petri B Chrobok NL Kubes P. Nucleation of platelets with blood-borne pathogens on Kupffer cells precedes other innate immunity and contributes to bacterial clearance. Nat Immunol (2013) 14(8):785–92. 10.1038/ni.2631 23770641 PMC4972575

[66] Cao TM Takatani T King MR. Effect of extracellular pH on selectin adhesion: theory and experiment. Biophys J (2013) 104(2):292–9. 10.1016/j.bpj.2012.12.005 23442851 PMC3552277

[67] Kuijper P Torres HG Van Der Linden J Lammers J Sixma J Koenderman L Platelet-dependent primary hemostasis promotes selectin and integrin-mediated neutrophil adhesion to damaged endothelium under flow conditions. Blood (1996) 87(8):3271–81. 8605343

[68] Page C Pitchford S. Neutrophil and platelet complexes and their relevance to neutrophil recruitment and activation. Int Immunopharmacol (2013) 17(4):1176–84. 10.1016/j.intimp.2013.06.004 23810443

[69] Assinger A Laky M Schabbauer G Hirschl AM Buchberger E Binder BR Efficient phagocytosis of periodontopathogens by neutrophils requires plasma factors, platelets and TLR2. J Thromb Haemost (2011) 9(4):799–809. 10.1111/j.1538-7836.2011.04193.x 21251195

[70] Grommes J Alard J-E Drechsler M Wantha S Mörgelin M Kuebler WM Disruption of platelet-derived chemokine heteromers prevents neutrophil extravasation in acute lung injury. Am J Respir Crit Care Med (2012) 185(6):628–36. 10.1164/rccm.201108-1533OC 22246174 PMC3326286

[71] Hartwig H Drechsler M Lievens D Kramp B von Hundelshausen P Lutgens E Platelet-derived PF4 reduces neutrophil apoptosis following arterial occlusion. Thromb Haemost (2014) 111(3):562–4 10.1160/TH13-08-0699 24258616

[72] Gawaz M Fateh-Moghadam S Pilz G Gurland HJ Werdan K. Platelet activation and interaction with leucocytes in patients with sepsis or multiple organ failure. Eur J Clin Invest (1995) 25(11):843–51. 10.1111/j.1365-2362.1995.tb01694.x 8582450

[73] Andrews RK Arthur JF Gardiner E Neutrophil extracellular traps (NETs) and the role of platelets in infection. Thromb Haemost (2014) 112(4):659–65 10.1160/TH14-05-0455 25144936

[74] Clark SR Ma AC Tavener SA McDonald B Goodarzi Z Kelly MM Platelet TLR4 activates neutrophil extracellular traps to ensnare bacteria in septic blood. Nat Med (2007) 13(4):463–9. 10.1038/nm1565 17384648

[75] McDonald B Urrutia R Yipp BG Jenne CN Kubes P. Intravascular neutrophil extracellular traps capture bacteria from the bloodstream during sepsis. Cell Host Microbe (2012) 12(3):324–33. 10.1016/j.chom.2012.06.011 22980329

[76] Kraemer BF Campbell RA Schwertz H Cody MJ Franks Z Tolley ND Novel anti-bacterial activities of β-defensin 1 in human platelets: suppression of pathogen growth and signaling of neutrophil extracellular trap formation. PLoS Pathog (2011) 7(11):e1002355. 10.1371/journal.ppat.1002355 22102811 PMC3213094

[77] Jenne CN Wong CH Zemp FJ McDonald B Rahman MM Forsyth PA Neutrophils recruited to sites of infection protect from virus challenge by releasing neutrophil extracellular traps. Cell Host Microbe (2013) 13(2):169–80. 10.1016/j.chom.2013.01.005 23414757

[78] Caudrillier A Kessenbrock K Gilliss BM Nguyen JX Marques MB Monestier M Platelets induce neutrophil extracellular traps in transfusion-related acute lung injury. J Clin Invest (2012) 122(7):2661. 10.1172/JCI61303 22684106 PMC3386815

[79] Rossaint J Herter JM Van Aken H Napirei M Döring Y Weber C Synchronized integrin engagement and chemokine activation is crucial in neutrophil extracellular trap-mediated sterile inflammation. Blood (2014) 123(16):2573–84. 10.1182/blood-2013-07-516484 24335230

[80] van Gils JM Zwaginga JJ Hordijk PL. Molecular and functional interactions among monocytes, platelets, and endothelial cells and their relevance for cardiovascular diseases. J Leukoc Biol (2009) 85(2):195–204. 10.1189/jlb.0708400 18948548

[81] Stephen J Emerson B Fox KA Dransfield I. The uncoupling of monocyte-platelet interactions from the induction of proinflammatory signaling in monocytes. J Immunol (2013) 191(11):5677–83. 10.4049/jimmunol.1301250 24133165

[82] Passacquale G Vamadevan P Pereira L Hamid C Corrigall V Ferro A. Monocyte-platelet interaction induces a pro-inflammatory phenotype in circulating monocytes. PLoS One (2011) 6(10):e25595. 10.1371/journal.pone.0025595 22022418 PMC3192052

[83] Scull CM Hays WD Fischer TH. Macrophage pro-inflammatory cytokine secretion is enhanced following interaction with autologous platelets. J Inflamm (Lond) (2010) 7:53. 10.1186/1476-9255-7-53 21067617 PMC2988777

[84] Halvorsen B Smedbakken LM Michelsen AE Skjelland M Bjerkeli V Sagen EL Activated platelets promote increased monocyte expression of CXCR5 through prostaglandin E2-related mechanisms and enhance the anti-inflammatory effects of CXCL13. Atherosclerosis (2014) 234(2):352–9. 10.1016/j.atherosclerosis.2014.03.021 24732574

[85] Xiang B Zhang G Guo L Li X-A Morris AJ Daugherty A Platelets protect from septic shock by inhibiting macrophage-dependent inflammation via the cyclooxygenase 1 signalling pathway. Nat Commun (2013) 4:2657. 10.1038/ncomms3657 24150174 PMC4217311

[86] Gudbrandsdottir S Hasselbalch HC Nielsen CH. Activated platelets enhance IL-10 secretion and reduce TNF-α secretion by monocytes. J Immunol (2013) 191(8):4059–67. 10.4049/jimmunol.1201103 24048901

[87] Li N. Platelet-lymphocyte cross-talk. J Leukoc Biol (2008) 83(5):1069–78. 10.1189/jlb.0907615 18198208

[88] Diacovo TG Catalina MD Siegelman MH Von Andrian UH. Circulating activated platelets reconstitute lymphocyte homing and immunity in L-selectin-deficient mice. J Exp Med (1998) 187(2):197–204. 10.1084/jem.187.2.197 9432977 PMC2212105

[89] Zamora C Cantó E Nieto JC Ortiz MA Diaz-Torné C Diaz-Lopez C Functional consequences of platelet binding to T lymphocytes in inflammation. J Leukoc Biol (2013) 94(3):521–9. 10.1189/jlb.0213074 23801652

[90] León-Ponte M Ahern GP O’Connell PJ. Serotonin provides an accessory signal to enhance T-cell activation by signaling through the 5-HT7 receptor. Blood (2007) 109(8):3139–46. 10.1182/blood-2006-10-052787 17158224 PMC1852236

[91] Katsounas A Schlaak JF Lempicki RA. CCL5: a double-edged sword in host defense against the hepatitis C virus. Int Rev Immunol (2011) 30(5–6):366–78. 10.3109/08830185.2011.593105 22053974

[92] Elzey BD Ratliff TL Sowa JM Crist SA. Platelet CD40L at the interface of adaptive immunity. Thromb Res (2011) 127(3):180–3. 10.1016/j.thromres.2010.10.011 21075431 PMC3073541

[93] Liu CY Battaglia M Lee SH Sun Q-H Aster RH Visentin GP. Platelet factor 4 differentially modulates CD4+ CD25+ (regulatory) versus CD4+ CD25-(nonregulatory) T cells. J Immunol (2005) 174(5):2680–6. 10.4049/jimmunol.174.5.2680 15728475

[94] Shi G Field DJ Ko KA Ture S Srivastava K Levy S Platelet factor 4 limits Th17 differentiation and cardiac allograft rejection. J Clin Invest (2014) 124(2):543. 10.1172/JCI71858 24463452 PMC3904624

[95] Danese S de la Motte C Reyes BMR Sans M Levine AD Fiocchi C. Cutting edge: T cells trigger CD40-dependent platelet activation and granular RANTES release: a novel pathway for immune response amplification. J Immunol (2004) 172(4):2011–5. 10.4049/jimmunol.172.4.2011 14764664

[96] Langer HF Daub K Braun G Schönberger T May AE Schaller M Platelets recruit human dendritic cells via Mac-1/JAM-C interaction and modulate dendritic cell function in vitro. Arterioscler Thromb Vasc Biol (2007) 27(6):1463–70. 10.1161/ATVBAHA.107.141515 17379836

[97] Hagihara M Higuchi A Tamura N Ueda Y Hirabayashi K Ikeda Y Platelets, after exposure to a high shear stress, induce IL-10-producing, mature dendritic cells in vitro. J Immunol (2004) 172(9):5297–303. 10.4049/jimmunol.172.9.5297 15100268

[98] Hamzeh-Cognasse H Cognasse F Palle S Chavarin P Olivier T Delézay O Direct contact of platelets and their released products exert different effects on human dendritic cell maturation. BMC Immunol (2008) 9(1):54. 10.1186/1471-2172-9-54 18817542 PMC2564901

[99] Nakanishi T Inaba M Inagaki-Katashiba N Tanaka A Vien PTX Kibata K Platelet-derived RANK ligand enhances CCL17 secretion from dendritic cells mediated by thymic stromal lymphopoietin. Platelets (2014):1–7. 10.3109/09537104.2014.920081 24867354

[100] Kissel K Berber S Nockher A Santoso S Bein G Hackstein H. Human platelets target dendritic cell differentiation and production of proinflammatory cytokines. Transfusion (2006) 46(5):818–27. 10.1111/j.1537-2995.2006.00802.x 16686850

[101] Panigrahi S Ma Y Hong L Gao D West XZ Salomon RG Engagement of platelet toll-like receptor 9 by novel endogenous ligands promotes platelet hyperreactivity and thrombosis. Circ Res (2013) 112(1):103–12. 10.1161/CIRCRESAHA.112.274241 23071157 PMC3537845

[102] Massberg S Brand K Grüner S Page S Müller E Müller I A critical role of platelet adhesion in the initiation of atherosclerotic lesion formation. J Exp Med (2002) 196(7):887–96. 10.1084/jem.20012044 12370251 PMC2194025

[103] Michelson AD Barnard MR Krueger LA Valeri CR Furman MI. Circulating monocyte-platelet aggregates are a more sensitive marker of in vivo platelet activation than platelet surface P-selectin studies in baboons, human coronary intervention, and human acute myocardial infarction. Circulation (2001) 104(13):1533–7. 10.1161/hc3801.095588 11571248

[104] Koenen RR von Hundelshausen P Nesmelova IV Zernecke A Liehn EA Sarabi A Disrupting functional interactions between platelet chemokines inhibits atherosclerosis in hyperlipidemic mice. Nat Med (2009) 15(1):97–103. 10.1038/nm.1898 19122657

[105] Strüßmann T Tillmann S Wirtz T Bucala R von Hundelshausen P Bernhagen J Platelets are a previously unrecognised source of MIF. Thromb Haemost (2013) 110(5):1004–13. 10.1160/TH13-01-0049 23846621

[106] Daub K Seizer P Stellos K Krämer BF Bigalke B Schaller M Oxidized LDL-activated platelets induce vascular inflammation. Semin Thromb Hemost (2010) 36(02):146–56. 10.1055/s-0030-1251498 20414829

[107] Burger PC Wagner DD. Platelet P-selectin facilitates atherosclerotic lesion development. Blood (2003) 101(7):2661–6. 10.1182/blood-2002-07-2209 12480714

[108] Lievens D Zernecke A Seijkens T Soehnlein O Beckers L Munnix IC Platelet CD40L mediates thrombotic and inflammatory processes in atherosclerosis. Blood (2010) 116(20):4317–27. 10.1182/blood-2010-01-261206 20705757 PMC2993630

[109] Mause SF Weber C. Microparticles protagonists of a novel communication network for intercellular information exchange. Circ Res (2010) 107(9):1047–57. 10.1161/CIRCRESAHA.110.226456 21030722

[110] Gawaz M Brand K Dickfeld T Pogatsa-Murray G Page S Bogner C Platelets induce alterations of chemotactic and adhesive properties of endothelial cells mediated through an interleukin-1-dependent mechanism. Implications for atherogenesis. Atherosclerosis (2000) 148(1):75–85. 10.1016/S0021-9150(99)00241-5 10580173

[111] Lindemann S Tolley ND Dixon DA McIntyre TM Prescott SM Zimmerman GA Activated platelets mediate inflammatory signaling by regulated interleukin 1β synthesis. J Cell Biol (2001) 154(3):485–90. 10.1083/jcb.200105058 11489912 PMC2196422

[112] Gawaz M Langer H May AE. Platelets in inflammation and atherogenesis. J Clin Invest (2005) 115(12):3378–84. 10.1172/JCI27196 16322783 PMC1297269

[113] Lievens D von Hundelshausen P Platelets in atherosclerosis. Thromb Haemost (2011) 106(5):827 10.1160/TH11-08-0592 22012554

[114] Simon DI. Inflammation and vascular injury: basic discovery to drug development. Circ J (2011) 76(8):1811–8. 10.1253/circj.CJ-12-0801 22785436 PMC4090145

[115] Simon DI Chen Z Xu H Li CQ Dong JF McIntire LV Platelet glycoprotein Ibα is a counterreceptor for the leukocyte integrin Mac-1 (CD11b/CD18). J Exp Med (2000) 192(2):193–204. 10.1084/jem.192.2.193 10899906 PMC2193258

[116] Manthey HD Zernecke A. Dendritic cells in atherosclerosis: functions in immune regulation and beyond. Thromb Haemost (2011) 106(5):772. 10.1160/TH11-05-0296 21901235

[117] Ricklin D Hajishengallis G Yang K Lambris JD. Complement: a key system for immune surveillance and homeostasis. Nat Immunol (2010) 11(9):785–97. 10.1038/ni.1923 20720586 PMC2924908

[118] Acosta J Qin X Halperin J. Complement and complement regulatory proteins as potential molecular targets for vascular diseases. Curr Pharm Des (2004) 10(2):203–11. 10.2174/1381612043453441 14754399

[119] Giannakopoulos B Passam F Rahgozar S Krilis SA. Current concepts on the pathogenesis of the antiphospholipid syndrome. Blood (2007) 109(2):422–30. 10.1182/blood-2006-04-001206 16985176

[120] Peerschke E Yin W Grigg S Ghebrehiwet B. Blood platelets activate the classical pathway of human complement. J Thromb Haemost (2006) 4(9):2035–42. 10.1111/j.1538-7836.2006.02065.x 16961611

[121] Peerschke EI Yin W Ghebrehiwet B Platelet mediated complement activation. Adv Exp Med Biol (2008) 632:81–91. 19025116 10.1007/978-0-387-78952-1_7 PMC2637649

[122] Peerschke EI Yin W Ghebrehiwet B. Complement activation on platelets: implications for vascular inflammation and thrombosis. Mol Immunol (2010) 47(13):2170–5. 10.1016/j.molimm.2010.05.009 20621693 PMC2904326

[123] Bäck J Huber Lang M Elgue G Kalbitz M Sanchez J Nilsson Ekdahl K Distinctive regulation of contact activation by antithrombin and C1-inhibitor on activated platelets and material surfaces. Biomaterials (2009) 30(34):6573–80. 10.1016/j.biomaterials.2009.07.052 19783299

[124] Hamad OA Nilsson PH Wouters D Lambris JD Ekdahl KN Nilsson B. Complement component C3 binds to activated normal platelets without preceding proteolytic activation and promotes binding to complement receptor 1. J Immunol (2010) 184(5):2686–92. 10.4049/jimmunol.0902810 20139276 PMC2953618

[125] Patzelt J Mueller K Breuning S Karathanos A Schleicher R Seizer P Expression of anaphylatoxin receptors on platelets in patients with coronary heart disease. Atherosclerosis (2015) 238(2):289–95. 10.1016/j.atherosclerosis.2014.12.002 25544179

[126] Verschoor A Langer HF Pan R Wang J Nardi M Li Z Crosstalk between platelets and the complement system in immune protection and disease. Thromb Haemost (2013) 110(5):910–9. 10.1160/TH13-02-0102 24008927

[127] McCarthy MJ Loftus IM Thompson MM Jones L London NJ Bell PR Angiogenesis and the atherosclerotic carotid plaque: an association between symptomatology and plaque morphology. J Vasc Surg (1999) 30(2):261–8. 10.1016/S0741-5214(99)70136-9 10436445

[128] von Birgelen C Klinkhart W Mintz GS Papatheodorou A Herrmann J Baumgart D Plaque distribution and vascular remodeling of ruptured and nonruptured coronary plaques in the same vessel: an intravascular ultrasound study in vivo. J Am Coll Cardiol (2001) 37(7):1864–70. 10.1016/S0735-1097(01)01234-7 11401124

[129] Tenaglia AN Peters KG Sketch MH Jr Annex BH. Neovascularization in atherectomy specimens from patients with unstable angina: implications for pathogenesis of unstable angina. Am Heart J (1998) 135(1):10–4. 10.1016/S0002-8703(98)70336-9 9453515

[130] Slevin M Turu MM Rovira N Luque A Baldellou M Krupinski J Identification of a ‘snapshot’ of co-expressed angiogenic markers in laser-dissected vessels from unstable carotid plaques with targeted arrays. J Vasc Res (2009) 47(4):323–35. 10.1159/000265566 20016206

[131] Kolodgie FD Gold HK Burke AP Fowler DR Kruth HS Weber DK Intraplaque hemorrhage and progression of coronary atheroma. N Engl J Med (2003) 349(24):2316–25. 10.1056/NEJMoa035655 14668457

[132] Virmani R Kolodgie FD Burke AP Finn AV Gold HK Tulenko TN Atherosclerotic plaque progression and vulnerability to rupture angiogenesis as a source of intraplaque hemorrhage. Arterioscler Thromb Vasc Biol (2005) 25(10):2054–61. 10.1161/01.ATV.0000178991.71605.18 16037567

[133] Hutter R Valdiviezo C Sauter BV Savontaus M Chereshnev I Carrick FE Caspase-3 and tissue factor expression in lipid-rich plaque macrophages evidence for apoptosis as link between inflammation and atherothrombosis. Circulation (2004) 109(16):2001–8. 10.1161/01.CIR.0000125526.91945.AE 15078795

[134] Fernández-Ortiz A Badimon JJ Falk E Fuster V Meyer B Mailhac A Characterization of the relative thrombogenicity of atherosclerotic plaque components: implications for consequences of plaque rupture. J Am Coll Cardiol (1994) 23(7):1562–9. 10.1016/0735-1097(94)90657-2 8195515

[135] Toschi V Gallo R Lettino M Fallon JT Gertz SD Ferna A Tissue factor modulates the thrombogenicity of human atherosclerotic plaques. Circulation (1997) 95(3):594–9. 10.1161/01.CIR.95.3.594 9024145

[136] Badimon JJ Lettino M Toschi V Fuster V Berrozpe M Chesebro JH Local inhibition of tissue factor reduces the thrombogenicity of disrupted human atherosclerotic plaques effects of tissue factor pathway inhibitor on plaque thrombogenicity under flow conditions. Circulation (1999) 99(14):1780–7. 10.1161/01.CIR.99.14.1780 10199872

[137] Day SM Reeve JL Pedersen B Farris DM Myers DD Im M Macrovascular thrombosis is driven by tissue factor derived primarily from the blood vessel wall. Blood (2005) 105(1):192–8. 10.1182/blood-2004-06-2225 15339841

[138] Bhattacharjee G Ahamed J Pedersen B El-Sheikh A Mackman N Ruf W Regulation of tissue factor-mediated initiation of the coagulation cascade by cell surface grp78. Arterioscler Thromb Vasc Biol (2005) 25(8):1737–43. 10.1161/01.ATV.0000173419.31242.56 15947236

[139] Sakakura K Nakano M Otsuka F Ladich E Kolodgie FD Virmani R. Pathophysiology of atherosclerosis plaque progression. Heart Lung Circ (2013) 22(6):399–411. 10.1016/j.hlc.2013.03.001 23541627

[140] Shah PK Falk E Badimon JJ Fernandez-Ortiz A Mailhac A Villareal-Levy G Human monocyte-derived macrophages induce collagen breakdown in fibrous caps of atherosclerotic plaques. Potential role of matrix-degrading metalloproteinases and implications for plaque rupture. Circulation (1995) 92(6):1565–9. 7664441

[141] Geng Y-J Libby P. Evidence for apoptosis in advanced human atheroma. Colocalization with interleukin-1 beta-converting enzyme. Am J Pathol (1995) 147(2):251. 7639325 PMC1869820

[142] Ehara S Kobayashi Y Yoshiyama M Shimada K Shimada Y Fukuda D Spotty calcification typifies the culprit plaque in patients with acute myocardial infarction an intravascular ultrasound study. Circulation (2004) 110(22):3424–9. 10.1161/01.CIR.0000148131.41425.E9 15557374

[143] Maldonado N Kelly-Arnold A Vengrenyuk Y Laudier D Fallon JT Virmani R A mechanistic analysis of the role of microcalcifications in atherosclerotic plaque stability: potential implications for plaque rupture. Am J Physiol Heart Circ Physiol (2012) 303(5):H619–28. 10.1152/ajpheart.00036.2012 22777419 PMC3468470

[144] Libby P Ridker PM Maseri A Inflammation and atherosclerosis. Circulation (2002) 105(9):1135–43 10.1161/hc0902.104353 11877368

[145] Ridker PM Thuren T Zalewski A Libby P. Interleukin-1β inhibition and the prevention of recurrent cardiovascular events: rationale and design of the canakinumab anti-inflammatory thrombosis outcomes study (CANTOS). Am Heart J (2011) 162(4):597–605. 10.1016/j.ahj.2011.06.012 21982649

[146] Ridker PM. Testing the inflammatory hypothesis of atherothrombosis: scientific rationale for the cardiovascular inflammation reduction trial (CIRT). J Thromb Haemost (2009) 7(1):332–9. 10.1111/j.1538-7836.2009.03404.x 19630828

[147] Gaziano JM Greenland P When should aspirin be used for prevention of cardiovascular events? JAMA (2014) 312(23):2503–4 10.1001/jama.2014.16047 25402671

[148] Verheugt FW Gersh BJ Aspirin beyond platelet inhibition. Am J Cardiol (2002) 90(1):39–41 10.1016/S0002-9149(02)02383-4 12088777

[149] Cyrus T Sung S Zhao L Funk CD Tang S Praticò D. Effect of low-dose aspirin on vascular inflammation, plaque stability, and atherogenesis in low-density lipoprotein receptor-deficient mice. Circulation (2002) 106(10):1282–7. 10.1161/01.CIR.0000027816.54430.96 12208806

[150] Tous M Ferré N Vilella E Riu F Camps J Joven J. Aspirin attenuates the initiation but not the progression of atherosclerosis in apolipoprotein E-deficient mice fed a high-fat, high-cholesterol diet. Basic Clin Pharmacol Toxicol (2004) 95(1):15–9. 10.1111/j.1742-7843.2004.pto950104.x 15245571

[151] Paul-Clark MJ van Cao T Moradi-Bidhendi N Cooper D Gilroy DW. 15-epi-lipoxin A4-mediated induction of nitric oxide explains how aspirin inhibits acute inflammation. J Exp Med (2004) 200(1):69–78. 10.1084/jem.20040566 15238606 PMC2213311

[152] Kopp E Ghosh S. Inhibition of NF-kappa B by sodium salicylate and aspirin. Science (1994) 265(5174):956–9. 10.1126/science.8052854 8052854

[153] Steinhubl SR Badimon JJ Bhatt DL Herbert J-M Lüscher TF. Clinical evidence for anti-inflammatory effects of antiplatelet therapy in patients with atherothrombotic disease. Vasc Med (2007) 12(2):113–22. 10.1177/1358863X07077462 17615799

[154] Ikeda Y Shimada K Teramoto T Uchiyama S Yamazaki T Oikawa S Low-dose aspirin for primary prevention of cardiovascular events in Japanese patients 60 years or older with atherosclerotic risk factors: a randomized clinical trial. JAMA (2014) 312(23):2510–20. 10.1001/jama.2014.15690 25401325

[155] Huynh K Atherosclerosis: low-dose aspirin failed to improve cardiovascular outcomes. Nat Rev Cardiol (2015) 12(1):3 10.1038/nrcardio.2014.194 25445143

[156] Li M Zhang Y Ren H Zhang Y Zhu X. Effect of clopidogrel on the inflammatory progression of early atherosclerosis in rabbits model. Atherosclerosis (2007) 194(2):348–56. 10.1016/j.atherosclerosis.2006.11.006 17156785

[157] Afek A Kogan E Maysel-Auslender S Mor A Regev E Rubinstein A Clopidogrel attenuates atheroma formation and induces a stable plaque phenotype in apolipoprotein E knockout mice. Microvasc Res (2009) 77(3):364–9. 10.1016/j.mvr.2009.01.009 19323972

[158] Quinn MJ Bhatt DL Zidar F Vivekananthan D Chew DP Ellis SG Effect of clopidogrel pretreatment on inflammatory marker expression in patients undergoing percutaneous coronary intervention. Am J Cardiol (2004) 93(6):679–84. 10.1016/j.amjcard.2003.11.048 15019868

[159] Ramadan R Dhawan SS Syed H Pohlel FK Binongo JN Ghazzal ZB Effects of clopidogrel therapy on oxidative stress, inflammation, vascular function, and progenitor cells in stable coronary artery disease. J Cardiovasc Pharmacol (2014) 63(4):369–74. 10.1097/FJC.0000000000000057 24336012 PMC4046585

[160] Azar RR Kassab R Zoghbi A Aboujaoudé S El-Osta H Ghorra P Effects of clopidogrel on soluble CD40 ligand and on high-sensitivity C-reactive protein in patients with stable coronary artery disease. Am Heart J (2006) 151(2):521.e1–4. 10.1016/j.ahj.2005.10.021 16442924

[161] Klinkhardt U Bauersachs R Adams J Graff J Lindhoff-Last E Harder S. Clopidogrel but not aspirin reduces P-selectin expression and formation of platelet-leukocyte aggregates in patients with atherosclerotic vascular disease. Clin Pharmacol Ther (2003) 73(3):232–41. 10.1067/mcp.2003.13 12621388

[162] Xiao Z Théroux P. Clopidogrel inhibits platelet-leukocyte interactions and thrombin receptor agonist peptide-induced platelet activation in patients with an acute coronary syndrome. J Am Coll Cardiol (2004) 43(11):1982–8. 10.1016/j.jacc.2003.10.071 15172401

[163] Waehre T Damås J Pedersen T Gullestad L Yndestad A Andreassen A Clopidogrel increases expression of chemokines in peripheral blood mononuclear cells in patients with coronary artery disease: results of a double-blind placebo-controlled study. J Thromb Haemost (2006) 4(10):2140–7. 10.1111/j.1538-7836.2006.02131.x 16856976

[164] Nagy B Jr Miszti-Blasius K Kerenyi A Clemetson KJ Kappelmayer J. Potential therapeutic targeting of platelet-mediated cellular interactions in atherosclerosis and inflammation. Curr Med Chem (2012) 19(4):518–31. 10.2174/092986712798918770 22204330

[165] Subbanagounder G Leitinger N Shih PT Faull KF Berliner JA. Evidence that phospholipid oxidation products and/or platelet-activating factor play an important role in early atherogenesis in vitro and in vivo inhibition by WEB 2086. Circ Res (1999) 85(4):311–8. 10.1161/01.RES.85.4.311 10455059

[166] Ewing MM de Vries MR Nordzell M Pettersson K de Boer HC van Zonneveld AJ Annexin A5 therapy attenuates vascular inflammation and remodeling and improves endothelial function in mice. Arterioscler Thromb Vasc Biol (2011) 31(1):95–101. 10.1161/ATVBAHA.110.216747 20947818

[167] Hamad OA Bäck J Nilsson PH Nilsson B Ekdahl KN. Platelets, complement, and contact activation: partners in inflammation and thrombosis. Adv Exp Med Biol (2012) 946:185–205. 10.1007/978-1-4614-0106-3_11 21948369

[168] Ricklin D Lambris JD. Complement-targeted therapeutics. Nat Biotechnol (2007) 25(11):1265–75. 10.1038/nbt1342 17989689 PMC2966895

[169] Ricklin D Lambris JD. Complement in immune and inflammatory disorders: therapeutic interventions. J Immunol (2013) 190(8):3839–47. 10.4049/jimmunol.1203200 23564578 PMC3623010

[170] Hill A Hillmen P Richards SJ Elebute D Marsh JC Chan J Sustained response and long-term safety of eculizumab in paroxysmal nocturnal hemoglobinuria. Blood (2005) 106(7):2559–65. 10.1182/blood-2005-02-0564 15985537

[171] Röth A Hock C Konik A Christoph S Dührsen U. Chronic treatment of paroxysmal nocturnal hemoglobinuria patients with eculizumab: safety, efficacy, and unexpected laboratory phenomena. Int J Hematol (2011) 93(6):704–14. 10.1007/s12185-011-0867-y 21611719

[172] Lindberg S Prognostic utility of the soluble CD40 ligand in acute coronary syndrome. Coron Artery Dis (2014) 25(7):548–9 10.1097/MCA.0000000000000155 25248136

[173] Setianto BY Hartopo AB Achadiono DN Gharini PP. Association between levels of circulating soluble CD40 ligand on admission and in-hospital events among acute coronary syndrome patients. Acta Med Indones (2011) 43(2):82–7. 21785169

[174] Pusuroglu H Akgul O Erturk M Uyarel H Bulut U Akkaya E Predictive value of elevated soluble CD40 ligand in patients undergoing primary angioplasty for ST-segment elevation myocardial infarction. Coron Artery Dis (2014) 25(7):558–64. 10.1097/MCA.0000000000000142 25004238

[175] Zhao W Zhang F Li Z Yu H Li Z Gao W. Soluble CD40 ligand is associated with angiographic severity of coronary artery disease in patients with acute coronary syndrome. Chin Med J (2014) 127(12):2218–21. 10.3760/cma.j.issn.0366-6999.20133159 24931231

[176] Pamukcu B Lip GY Snezhitskiy V Shantsila E. The CD40-CD40L system in cardiovascular disease. Ann Med (2011) 43(5):331–40. 10.3109/07853890.2010.546362 21244217

[177] Ridker PM Buring JE Rifai N. Soluble P-selectin and the risk of future cardiovascular events. Circulation (2001) 103(4):491–5. 10.1161/01.CIR.103.4.491 11157711

[178] Kisucka J Chauhan AK Zhao B-Q Patten IS Yesilaltay A Krieger M Elevated levels of soluble P-selectin in mice alter blood-brain barrier function, exacerbate stroke, and promote atherosclerosis. Blood (2009) 113(23):6015–22. 10.1182/blood-2008-10-186650 19349621 PMC2700332

